# Combined arthroscopic rotator cuff repair leads to better clinical outcomes than isolated removal of calcific deposits for shoulder calcific tendinitis: A 2- to 5-year follow-up study

**DOI:** 10.3389/fsurg.2022.912779

**Published:** 2022-08-17

**Authors:** Long Pang, Tao Li, Yinghao Li, Yuanyinuo Cao, Jian Li, Jing Zhu, Xin Tang

**Affiliations:** ^1^ Department of Orthopedics, Orthopedic Research Institute, West China Hospital, Sichuan University, Chengdu, China; ^2^ West China Medical School, Sichuan University, Chengdu, China; ^3^ Department of Respiratory and Critical Care Medicine, West China Hospital, Sichuan University, Chengdu, China

**Keywords:** shoulder, calcific tendinitis, arthroscopic, rotator cuff repair, debridement

## Abstract

**Purpose:**

The optimal treatment procedure for shoulder calcific tendinitis (CT) remains controversial. This study aimed to assess the efficacy of arthroscopic treatment for CT, and to compare the clinical outcomes following combined rotator cuff repair and isolated removal of calcific deposits.

**Methods:**

This retrospective cohort study included 46 patients (47 shoulders) with confirmed shoulder CT, and the diameter of the calcific deposit was over 1 cm. All patients suffered from CT for a mean period of 17.82 months and had a poor response to conservative treatment. With 12 males and 34 females included, the mean age was 53.94 years. After failed conservative treatment, 23 shoulders underwent combined rotator cuff repair (repair group), and 24 shoulders underwent isolated removal of calcific deposits (debridement group). The clinical outcomes were evaluated at baseline, 3, 6, and 12 months after the surgery and annually thereafter. The efficacy measures included the visual analog scale (VAS) pain score, American Shoulder and Elbow Surgeons (ASES) score, University of California at Los Angeles (UCLA) score and radiographic outcomes.

**Results:**

Remarkable improvement in clinical outcomes at the final follow-up (2- to 5-year) compared with those at baseline were observed (*p *< 0.0001 for all outcomes). Compared with isolated removal of calcific deposits, combined rotator cuff repair led to worse postoperative 3- and 6-month VAS (*p* = 0.004 and *p* = 0.026, respectively), and 3-month ASES scores (*p* = 0.012). However, better VAS (*p* = 0.035 and *p* = 0.007, respectively) and ASES (*p* = 0.034 and *p* = 0.020, respectively) scores at 24-month and final follow-up were found in the repair group. All these differences reached the minimal clinical important difference (MCID). MRI scans at the final follow-up showed significantly better outcomes in patients with rotator cuff repair (*p* = 0.021).

**Conclusions:**

Arthroscopic removal of calcific deposits is safe and effective for treating CT. Compared with isolated debridement, combined rotator cuff repair led to worse short-term (<12 months) but better medium- (12–48 months) to long-term (≥48 months) improvements in pain, function and integrity of tendons.

## Introduction

Rotator cuff calcific tendinitis (CT) is a common musculoskeletal disorder with an incidence rate of 2.7%–22% (1, 2). Normally, CT tends to occur in women aged 30–60 years (3). It is characterized by pain and calcific deposits in the rotator cuff or synovial tissue, with the supraspinatus tendon being the most commonly affected (80%) (4, 5). The etiopathogenesis of CT is still unclear, and the main theories include chronic degeneration, ischemia, incorrect differentiation of tendon stem cells into bone cells, tendon hypoxia, and hormonal changes (6–9). Uhthoff et al. (10) described CT as a self-limiting disease consisting of precalcific, calcific and postcalcific stages. The calcific stage is further subdivided into formative, resting and resorption phases. Usually, there are no apparent symptoms during the resting phase, while patients could experience mild pain during the formative phase and severe pain during the resorption phase (10).

Conservative management is the initial treatment for previously untreated CT. This includes nonsteroidal anti-inflammatory drugs (NSAIDs), physical therapy, subacromial injections, extracorporeal shockwave therapy (ESWT), ultrasound-guided needling (UGN) or percutaneous irrigation of calcific tendinopathy (US-PICT), which have been reported to yield benefits to most patients with CT (7, 11–14). However, if symptoms persist over 6 months after the start of conservative treatment, open or arthroscopic removal of the deposit should be considered (7). Arthroscopy is recommended for similar results but has lower morbidity rates and earlier recovery than open surgery (14).

After removal of calcific deposits with various shapes and sizes, defects may occur in the rotator cuff. It remains controversial whether the defect should be repaired. A recent study (15) comparing debridement (isolated removal of calcific deposits) with or without tendon repair independent of the size and shape of the deposit reported favourable outcomes with combined repair. However, in clinical practice, after arthroscopic removal of the deposit, combined rotator cuff repair would usually be performed when a relatively large defect was created, while isolated debridement would be enough for most relatively smaller defects. In this retrospective study, we aimed to evaluate the clinical and structural outcomes following arthroscopic treatment for CT at 2- to 5- year follow-up. Furthermore, subgroup analysis was performed to find out whether there was significant difference between combined rotator cuff repair and isolated removal of calcific deposits. It is hypothesized that arthroscopic treatment would show excellent results for this cohort patients with CT. As for subgroup analysis, combined rotator cuff repair would yield better improvements than isolated removal of calcific deposits at medium- to long-term clinical outcomes.

## Methods

This was a retrospective case series of prospectively collected clinical data. The study was approved by the Ethics Committee of West China Hospital, and was performed in accordance with the Declaration of Helsinki. All patients provided informed consent prior to enrollment in the study.

### Study population

**Figure 1:**
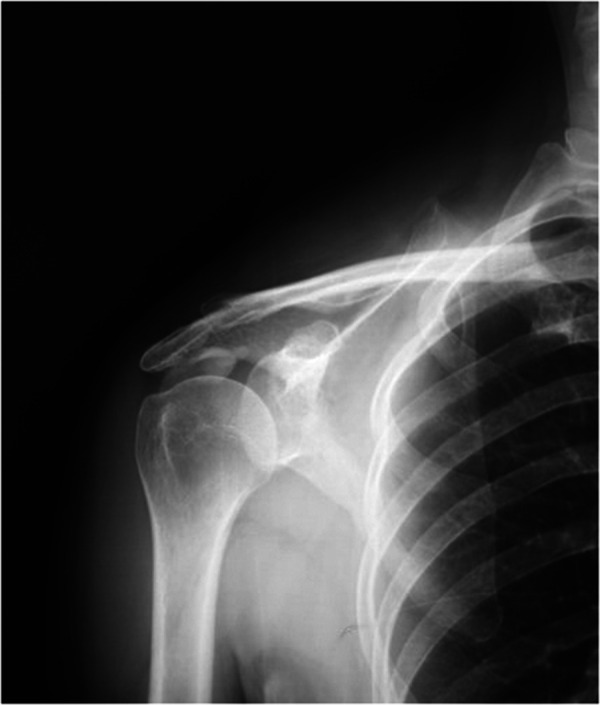
X-ray (front) showed clear calcific deposits in the target shoulder.

**Figure 2:**
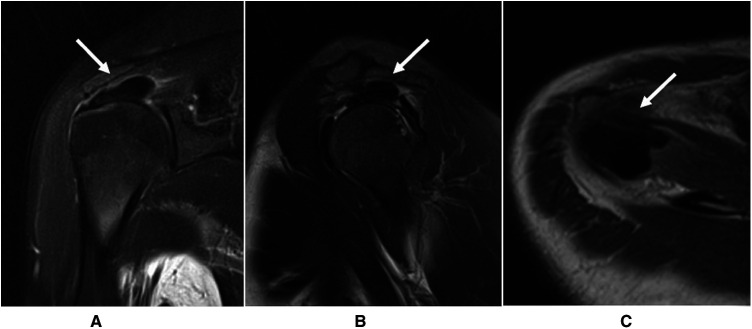
MRI showed the calcific deposits in the target shoulder in in the (**A**) oblique coronal; (**B**) sagittal; and (**C**) horizontal plane.

The inclusion criteria of this study were as follows: (1) absolute x-ray (Figure 1) and MRI (Figure 2) evidence of CT with a diameter of the deposit >1 cm (grade II and III according to Bosworth classification) (1, 16, 17); (2) patients with more than 60 mm on a 100-mm visual analog scale (VAS) for night pain (during the resorption phase) (18, 19); and (3) poor response to conservative treatments for at least 6 months (19). The exclusion criteria were as follows: (1) systemic diseases or other diseases of the affected shoulder; (2) history of previous surgical procedures in the affected shoulder; or (3) the follow-up period <2 years.

Between January 2015 to August 2020, a total of 63 patients who underwent surgery for rotator cuff CT in our department were screened. Eleven patients were excluded: six patients did not undergo conservative treatments for more than 6 months, four patients had full rotator cuff tears before removal of calcific deposits, which were confirmed by arthroscopic examination, and one patient had a concomitant immune disease. A total of 52 patients met the inclusion criteria, and 46 of those patients were followed for a minimum of 2 years (follow-up rate: 88.5%). All surgical procedures were performed by the same experienced surgeon.

### Surgical procedure

All surgical procedures were performed with the patient in a lateral decubitus position after general anesthesia. First, arthroscopy through the routine posterior portal was used to evaluate the glenohumeral joint. Then, the routine anterior upper portal was established under arthroscopic guidance. Intra-articular pathologies were identified and managed, as required. Subsequently, the scope was moved to the surface of the bursa for further evaluation, and subacromial decompression was performed if evidence of impingement was observed. Then, under needle guidance, the routine lateral portal was made, and the scope was moved to this portal for an outlet view.

**Figure 3:**
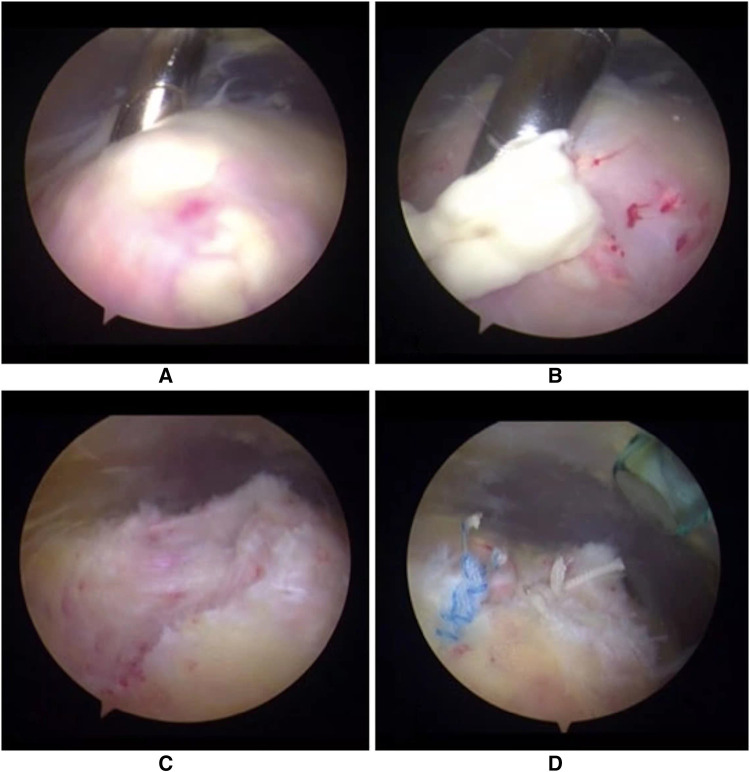
The calcific materials were found in (**A**) a more superficial location; (**B**) being removed; (**C**) a rotator cuff defect occurred after removal of calcific deposits; (**D**) the rotator cuff defect was repaired using one double-loaded anchor.

Prior to the operation, the supraspinatus outlet view was thoroughly evaluated to locate the calcific lesion in the rotator cuff tendon. Usually, calcific materials can be easily identified because calcific lesions are generally in a superficial location (Figure 3A). In some cases, it was difficult to visualize any pathologies on the outer surfaces of the tendons, so exploration of the supraspinatus tendon was performed using a spinal needle to identify the calcific materials. A spinal needle was introduced into the supraspinatus tendon percutaneously to locate the calcific deposits. Once the calcific lesion was located, an combined lateral portal was normally established, and a small longitudinal incision (measuring no more than 1.5 cm) parallel to the rotator cuff tendon on the synovial side was made using a no.11 sharp blade so that all of the calcific materials could be removed. For the three calcific lesions in the subscapularis tendon, a combined anterolateral portal was established when we located the calcific lesion, and a small longitudinal incision (no more than 1.5 cm) parallel to the subscapularis tendon was made.

Patients with signs of subacromial impingement who were arthroscopically confirmed underwent release of the coracoacromial ligament and flattening of the anterior-inferior surface of the acromion. This was performed with a combination of electrocautery and shaver to remove bursal tissue and define the lateral border and undersurface of the acromion. A motorized bur was then applied to remove spurs until the undersurface of the acromion was viewed as flat from the lateral portal.

In all 46 patients with 47 affected shoulders, 18 lesions were identified directly under arthroscopy, and the other 29 lesions were confirmed by spinal needles. All visible calcifications were removed by irrigation or debridement and were sent for further pathological assessments (Figure 3B). After complete removal of the calcification and the debridement of serious degenerative changes in the surrounding tissue, a rotator cuff defect developed in most cases (Figure 3C).

When the rotator cuff defect was considered to be a relatively large defect or the tendon was a full-thickness tear after removal of calcific deposits, repair was performed using one or two suture anchors or side-to-side stitches, depending on the size and shape of the defect (Figure 3D). Suture anchor repair was performed when there were relatively large defects (>1 cm) and partial thickness tears with an Ellman grade higher than III after the removal of calcific materials, and one stitch side-to-side repair was performed in partial tears with an Ellman grade of II to prevent the progression of the rotator cuff tears. In cases with minimal damage (an Ellman grade of I or lower) of the rotator cuff after the removal of the calcific deposits, only debridement was performed.

### Postoperative management and assessment

All patients received an intramuscular injection of pethidine and oral NSAIDs after surgery. A shoulder abduction brace was used for 4–6 weeks after the surgery for patients who underwent combined rotator cuff repair, and then, active range of motion and strengthening exercises were started gradually. All patients who underwent rotator cuff repair were prescribed passive range of motion exercises 2 weeks after surgery (20). Only supervised passive and active exercises as tolerated were allowed during the first 6 weeks for patients who underwent isolated debridement.

All patients underwent clinical follow-ups at intervals of 3, 6, and 12 months after the surgery and annually thereafter. All clinical efficacy measures, including the ASES scores, UCLA scores, and VAS scores at night, were determined by two independent blinded surgeons. The minimal clinical important difference (MCID) was set as at least a 14-mm change (on a 100-mm scale) in VAS, a 4-point change in ASES, a 3.5-point change in UCLA (21). The radiographic data were reviewed by two experienced physicians from our hospital imaging center.

### Statistical analysis

Statistical analyses were performed by SPSS 22.0 software (SPSS Inc., Chicago, IL, United States). Continuous data were presented as the mean ± SD. Descriptive data were presented as frequencies and percentages. The Kolmogorov–Smirnov test was used to test specifications for normal distribution. Differences between pre- and postoperative mean ASES, UCLA and VAS scores were analysed by paired *t* tests. Independent *t* tests were used to compare these outcomes in subgroup analysis if the distribution was normal, and the Mann–Whitney *U* test was applied when the distribution was not normal. To determine differences in paired observations within one group, the Wilcoxon test was applied. Categorical variables were analyzed using the chi-square test or Fisher exact test. A *p* value of less than 0.05 was considered significant.

### Sample size calculation

In this study, the 0- to 100-mm VAS was used as the primary outcome measure. A difference of 14-mm VAS was defined as the MCID between the two groups. With an assumed SD of 20 mm, we computed that a sample size of 20 patients allocated in each treatment group would achieve a power of 90% to detect a 14-mm difference. The statistical level of significance was set at 0.05.

## Results

A total of 46 patients (47 shoulders) met the inclusion criteria. The detailed baseline characteristics for all 46 patients are shown in Table 1. Combined rotator cuff repair (repair group) was performed in 23 shoulders (49%), and isolated removal of calcific deposits (debridement group) was performed in 24 shoulders (51%). All the baseline data of the two subgroups were comparable, except for the size of deposits measured.

**Table 1:** Demographic baseline data of the included patients (*n* = 46).

	Total (*n* = 46)	Repair group (*n* = 22)	Debridement group (*n* = 24)	*p* value^b^
Age, (mean ± SD), year	53.94 ± 5.23	54.09 ± 5.88	53.83 ± 4.99	NS
Sex, male/female	12/34	6/16	6/18	NS
Affected side^a^, right/left	32/15	15/8	17/7	NS
Diabetes mellitus, *n*	11	6	5	NS
Steroid injection before surgery^a^, *n*	16	9	7	NS
Affected tendon^a^, *n*				
Supraspinatus	35	15	20	NS
Infraspinatus	6	4	2	NS
Both above	3	2	1	NS
Subscapularis	3	2	1	NS
Concomitant PRCT^a^, *n*				
Elman grade I	13	8	5	NS
Elman grade II	6	4	2	NS
Gartner classification, *n*				
Type I (sharp or dense)	35	16	19	NS
Type II (poorly defined sharp or dense contours)	9	5	4	NS
Type III (poorly defined, transparent)	3	2	1	NS
Bosworth classification, *n*				
Bosworth II (1–1.5 cm)	25	3	22	*p *< 0.0001
Bosworth III (>1.5 cm)	22	20	2	*p *< 0.0001
Subacromial decompression, *n*	28	15	13	NS
Duration of symptoms^a^, (mean ± SD), m	17.82 ± 5.12	18.45 ± 5.67	17.05 ± 4.95	NS
Follow-up time^a^, (mean ± SD), m	44.53 ± 16.9	43.38 ± 16.85	45.65 ± 17.2	NS

PTRCT, partial-thickness rotator cuff tear; SD, standard deviation; NS, not significant.

^a^ 47 shoulders.

^b^ Differences between the baseline data of two subgroup patients.

During the follow-up, no recurrences, infections, subcutaneous hematomas or other complications occurred, and no combined operations were required for any patient. Six patients (four in the repair group, and two in the debridement group) who did not have any motion limitations prior to surgery developed shoulder stiffness, which was resolved within 6 months by individualized rehabilitation.

**Figure 4:**
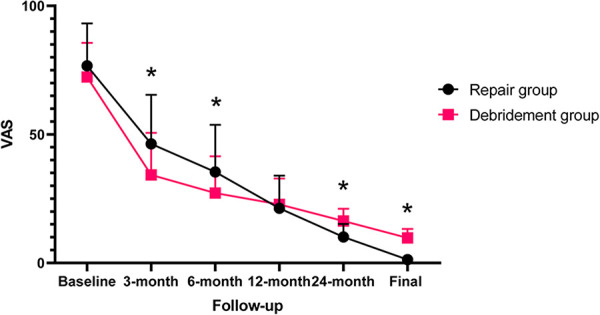
Postoperative VAS scores of the two subgroups at different follow-up time.

**Figure 5:**
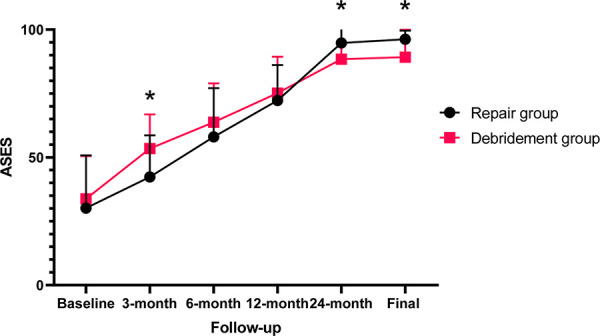
Postoperative ASES scores of the two subgroups at different follow-up time.

**Figure 6:**
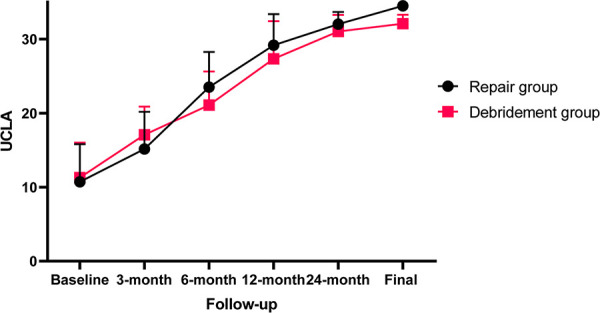
Postoperative UCLA scores of the two subgroups at different follow-up time.

All outcomes improved significantly (*p *< 0.0001) after arthroscopic removal of calcific deposits at the final follow-up (Table 2). The VAS and function scores at baseline of the two subgroups were comparable. Compared with patients who underwent isolated removal of calcific deposits, those who underwent combined rotator cuff repair achieved worse VAS scores at the 3-month (*p* = 0.004) and 6-month follow-ups (*p* = 0.026), but had lowerVAS scores at the 24-month (*p* = 0.035) and final follow-ups (*p* = 0.007). In addition, combined rotator cuff repair provided a lower ASES score at the 3-month follow-up (*p* = 0.012), but significantly higher ASES scores at the 24-month (*p* = 0.034) and final follow-ups (*p* = 0.020). All these differences in VAS and ASES scores between the two groups reached the MCID. There were no statistically significant differences between the two subgroups in VAS score at the 12-month follow-up, ASES score at the 6- and 12-month follow-ups or UCLA score at any period of follow-up (Figures 4–6).

**Table 2:** Pre-operative and post-operative VAS, ASES, and UCLA scores.

Outcomes	Pre-operative	3-month	6-month	12-month	24-month	Final	*p* value^a^
VAS	74.36 ± 14.20	39.80 ± 17.73	31.53 ± 15.80	21.58 ± 11.65	13.24 ± 4.90	5.14 ± 2.75	<0.0001
ASES	31.76 ± 18.10	47.32 ± 14.73	60.13 ± 17.35	74.18 ± 14.10	91.55 ± 8.20	94.71 ± 3.05	<0.0001
UCLA	11.03 ± 5.07	16.74 ± 4.52	22.48 ± 4.65	28.43 ± 4.80	31.02 ± 1.91	33.78 ± 0.70	<0.0001

VAS, visual analogue score; ASES, American Shoulder Elbow Scale; UCLA, University of California and Los Angeles shoulder score.

^a^ Differences between pre-operative and final data analysed by paired *t* tests.

**Figure 7:**
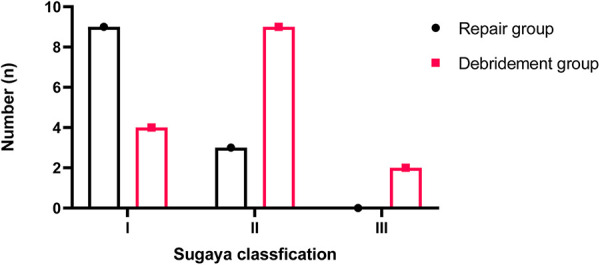
Tendon integrity detected by MRI scans according to the Sugaya classification at final follow-up.

Plain radiographs obtained 1 day after surgery revealed complete removal of calcific deposits in all patients. At the final follow-up, the MRI (12 shoulders in the repair group, 15 shoulders in the debridement group) results confirmed that all patients demonstrated complete resorption without recurrence. MRI examinations of 9 and 3 shoulders in the repair group showed a Sugaya I and Sugaya II classification (22), while 4, 9, and 2 shoulders in the debridement group showed Sugaya I, II and III classifications respectively (Figure 7). These differences were significantly different (*p* = 0.021).

## Discussion

This study assessed the efficacy of arthroscopic treatment of CT, and further explored whether there were differences between combined rotator cuff repair and isolated removal of calcific deposits. The present study revealed that arthroscopic removal of calcific deposits could yield satisfying clinical outcomes in pain reduction and functional recovery at medium- to long-term follow-up. The most important new finding of this study was that combined rotator cuff repair after removal of deposits led to worse short-term (≤6 months) but better medium- to long-term (≥24 months) pain relief and functional improvement than isolated removal of calcific deposits. Moreover, rotator cuff repair provided superior medium- to long-term structural integrity of the rotator cuff compared with isolated removal of calcific deposits.

Prospective randomized trials investigating the effect of arthroscopic treatment on clinical outcomes in patients with CT are lacking. Several studies have reported excellent results with arthroscopic removal of calcific deposits (23–28), which are consistent with our findings. However, debates over the optimal arthroscopic procedure for CT are still ongoing.

First, whether the calcific deposits should be totally removed remains controversial. Some surgeons claimed that complete removal was unnecessary because partial removal also achieved good results (29, 30). Moreover, partial removal can not only trigger the resorption of residual deposits but also better preserve the tendon (30). In contrast, some studies have shown better outcomes of the complete removal of deposits, and a negative correlation between the functional outcomes and the amount of the remaining vestigial materials (26, 31–32). In this study, all patients underwent complete removal of calcific deposits with or without combined rotator cuff repair. Although a comparison between complete removal and partial removal was not made, all patients enrolled in this study had satisfying outcomes and no recurrences at a minimum of 2 years of follow-up. Consistent with these previous outcomes, our data supported that complete removal might be superior to partial removal, which needs to be verified by further prospective randomized studies

After complete removal of the deposits, a defect of the rotator cuff usually remains. There is debate regarding whether the defect should be repaired. Many authors have considered the suturing of residual tendon lesions unnecessary, because the nature of CT is self-healing (7). Some studies have shown good results of arthroscopic isolated removal of calcific deposits without rotator cuff repair, but irregular remnants within the tendon, worse function and higher rates of partial rotator cuff tears compared with the contralateral shoulder were also reported (23, 30). In another study by Yoo et al. (32), no significant differences were observed in constant or ASES scores between the repair group and debridement groups. However, suture anchor repair was performed in cases with extensive defects (repair group), while other patients with minimal defects underwent either side-to-side repair or isolated removal of calcific deposits (debridement group). Under this condition, their conclusions should be interpreted with caution.

Conversely, some authors have recommended suture repair of the residual tendon lesions when the remaining defect was observed (7, 14). Porcellini et al. (26) analyzed 63 patients who underwent arthroscopic debridement with combined repair of the tendon defect. They recommended suturing of the defects to prevent further propagation of the tear and to facilitate early rehabilitation. Hashiguchi et al. (33) evaluated clinical and radiological outcomes of arthroscopic treatment for 37 patients with refractory rotator cuff calcific tendinitis. After accurately determining the size and location of calcific deposits by radiographs and three-dimensional computed tomography (CT), they removed calcific deposits as much as possible and sutured tendons with a side-to-side repair with strong sutures. Satisfactory pain reduction was reported in all patients, while residual calcific deposits were detected by postoperative radiographs in only three patients at final follow-up (mean 30.4 months). Recently, Lorbach et al. (15) compared the structural and clinical results after arthroscopic rotator cuff repair or isolated debridement. With 44 patients enrolled and a mean follow-up of 58.4 months, all patients were satisfied with the outcomes. Significantly better results in the repair group regarding the Constant score; the ASES score; the Isolated Shoulder Test; and the numerical rating scales for pain, function, and satisfaction were found. Postoperative tendon integrity showed 80% of the shoulders with a Sugaya type I classification in the rotator cuff repair group and 64% of the shoulders with a Sugaya type II classification in the debridement group (*p* = 0.021). These findings were highly consistent with those of this present study. Different from these studies, we compared not only short-term but also medium- to long-term outcomes between larger defects treated by suturing and smaller defects treated by debridement, which is closer to clinical practice.

Our final results suggest that arthroscopic treatment of CT is highly effective, and combined rotator cuff repair is beneficial, especially for more extensive defects. Although the preoperative sizes of deposits were not comparable between the two groups, patients with larger tendon defects in the repair group achieved significantly better clinical outcomes at final follow-up compared with those in the debridement group. Moreover, MRI scans at the final follow-up showed 75% of the shoulders with a Sugaya type I classification in the repair group, while the debridement group had 60% of shoulders with a Sugaya type II classification. It is worth mentioning that, many patients in the repair group were reluctant to receive an MRI at the final follow-up because they had fully recovered. These results strongly support suturing the defects of the rotator cuff after total removal of the calcific deposits.

However, combined repair also led to worse pain relief at the 3- and 6-month follow-ups, along with lower ASES scores at the 3-month follow-up. We consider it reasonable based on two assumptions. First, patients in the repair group had relative immobilization until protected passive range of motion exercises started at 2 weeks after surgery and active exercises started at 4–6 weeks after surgery (20), while those in the debridement group started supervised passive and active exercises immediately after surgery, resulting in a possible higher risk of developing shoulder stiffness after repair. The incidences of postoperative shoulder stiffness were approximately 17% (4/23) in the repair group and 8% (2/24) in the debridement group. Second, patients who underwent rotator cuff repair had relatively larger tendon defects, so it took more time to achieve tendon healing.

Several limitations of our analysis should be noted. First, this was a retrospective study without randomization, but the clinical data were prospectively collected. As a result, the independent reviewers were blinded. Second, although most baseline data of the two subgroups were comparable, the sizes of the calcific deposits were not comparable. However, patients with larger deposits achieved better outcomes at final follow-up, indicating that improvements were mainly due to combined rotator cuff repair. Third, the number of final MRI scans was limited due to high expenses and long waiting periods. Last, rehabilitation courses after surgery varied from groups, which could have an influence on postoperative clinical outcomes.

## Conclusion

Arthroscopic removal of calcific deposits is a safe and effective treatment for patients with CT. Patients who underwent combined repair of rotator cuff defects had worse short-term (≤6 months) but better medium to long-term (>24 months) improvement in pain and function than those underwent isolated removal of calcific deposits. In addition, combined repair was related to better integrity of tendons. This conclusion should be interpreted with caution, and further prospective randomized studies with lager sample sizes are needed to confirm the findings of this study.

## Data Availability

The original contributions presented in the study are included in the article/Supplementary Material, further inquiries can be directed to the corresponding authors.

## References

[1] Bosworth BM. Calcium deposits in the shoulder and subacromial bursitis: a survey of 12,122 shoulders. J Am Med Assoc. (1941) 116(22):2477–82. 10.1001/jama.1941.02820220019004

[2] Depalma AF Kruper JS. Long-term study of shoulder joints afflicted with and treated for calcific tendinitis. Clin Orthop. (1961) 20:61–72. 13721957

[3] ElShewy MT. Calcific tendinitis of the rotator cuff. World J Orthop. (2016) 7(1):55–60. 10.5312/wjo.v7.i1.55 26807357 PMC4716572

[4] Bureau NJ. Calcific tendinopathy of the shoulder. Semin Musculoskelet Radiol. (2013) 17(1):80–4. 10.1055/s-0033-1333941 23487339

[5] Becciolini M Bonacchi G Galletti S. Intramuscular migration of calcific tendinopathy in the rotator cuff: ultrasound appearance and a review of the literature. J Ultrasound. (2016) 19(3):175–81. 10.1007/s40477-016-0202-9 27635162 PMC5005209

[6] Gohlke F. Early European contributions to rotator cuff repair at the turn of the 20th century. J Shoulder Elb Surg. (2011) 20(3):352–7. 10.1016/j.jse.2011.01.001 21397789

[7] Sansone V Maiorano E Galluzzo A Pascale V. Calcific tendinopathy of the shoulder: clinical perspectives into the mechanisms, pathogenesis, and treatment. Orthop Res Rev. (2018) 10:63–72. 10.2147/ORR.S138225 30774461 PMC6209365

[8] Rui YF Lui PP Chan LS Chan KM Fu SC Li G. Does erroneous differentiation of tendon-derived stem cells contribute to the pathogenesis of calcifying tendinopathy? Chin Med J. (2011) 124(4):606–10. 10.3760/cma.j.issn.0366-6999.2011.04.022 21362289

[9] Harvie P Pollard TC Carr AJ. Calcific tendinitis: natural history and association with endocrine disorders. J Shoulder Elb Surg. (2007) 16(2):169–73. 10.1016/j.jse.2006.06.007 17188907

[10] Uhthoff HK Loehr JW. Calcific tendinopathy of the rotator cuff: pathogenesis, diagnosis, and management. J Am Acad Orthop Surg. (1997) 5(4):183–91. 10.5435/00124635-199707000-00001 10797220

[11] Daecke W Kusnierczak D Loew M. Long-term effects of extracorporeal shockwave therapy in chronic calcific tendinitis of the shoulder. J Shoulder Elb Surg. (2002) 11(5):476–80. 10.1067/mse.2002.126614 12378167

[12] Lee SY Cheng B Grimmer-Somers K. The midterm effectiveness of extracorporeal shockwave therapy in the management of chronic calcific shoulder tendinitis. J Shoulder Elb Surg. (2011) 20(5):845–54. 10.1016/j.jse.2010.10.024 21232988

[13] de Witte PB Kolk A Overes F Nelissen R Reijnierse M. Rotator cuff calcific tendinitis: ultrasound-guided needling and lavage versus subacromial corticosteroids: five-year outcomes of a randomized controlled trial. Am J Sports Med. (2017) 45(14):3305–14. 10.1177/0363546517721686 28898104

[14] Hurt G Baker CL Jr. Calcific tendinitis of the shoulder. Orthop Clin North Am. (2003) 34(4):567–75. 10.1016/S0030-5898(03)00089-0 14984196

[15] Lorbach O Haupert A Berger C Brockmeyer M. Clinical and structural results of rotator cuff repair compared with rotator cuff debridement in arthroscopic treatment of calcifying tendinitis of the shoulder. Am J Sports Med. (2021) 49(12):3196–201. 10.1177/03635465211037690 34528841

[16] Gosens T Hofstee DJ. Calcifying tendinitis of the shoulder: advances in imaging and management. Curr Rheumatol Rep. (2009) 11(2):129–34. 10.1007/s11926-009-0018-0 19296885

[17] Albano D Coppola A Gitto S Rapisarda S Messina C Sconfienza LM. Imaging of calcific tendinopathy around the shoulder: usual and unusual presentations and common pitfalls. Radiol Med. (2021) 126(4):608–19. 10.1007/s11547-020-01300-0 33151457 PMC8007494

[18] Sansone V Consonni O Maiorano E Meroni R Goddi A. Calcific tendinopathy of the rotator cuff: the correlation between pain and imaging features in symptomatic and asymptomatic female shoulders. Skeletal Radiol. (2016) 45(1):49–55. 10.1007/s00256-015-2240-3 26306389

[19] Merolla G Singh S Paladini P Porcellini G. Calcific tendinitis of the rotator cuff: state of the art in diagnosis and treatment. J Orthop Traumatol. (2016) 17(1):7–14. 10.1007/s10195-015-0367-6 26163832 PMC4805635

[20] Thigpen CA Shaffer MA Gaunt BW Leggin BG Williams GR Wilcox RB. The American society of shoulder and elbow therapists’ consensus statement on rehabilitation following arthroscopic rotator cuff repair. J Shoulder Elb Surg. (2016) 25(4):521–35. 10.1016/j.jse.2015.12.018 26995456

[21] Jones IA Togashi R Heckmann N Vangsness CT Jr. Minimal clinically important difference (MCID) for patient-reported shoulder outcomes. J Shoulder Elb Surg. (2020) 29(7):1484–92. 10.1016/j.jse.2019.12.033 32249146

[22] Sugaya H Maeda K Matsuki K Moriishi J. Functional and structural outcome after arthroscopic full-thickness rotator cuff repair: single-row versus dual-row fixation. Arthroscopy. (2005) 21(11):1307–16. 10.1016/j.arthro.2005.08.011 16325080

[23] Balke M Bielefeld R Schmidt C Dedy N Liem D. Calcifying tendinitis of the shoulder: midterm results after arthroscopic treatment. Am J Sports Med. (2012) 40(3):657–61. 10.1177/0363546511430202 22156173

[24] Lorbach O Kusma M Pape D Kohn D Dienst M. Influence of deposit stage and failed ESWT on the surgical results of arthroscopic treatment of calcifying tendonitis of the shoulder. Knee Surg Sports Traumatol Arthrosc. (2008) 16(5):516–21. 10.1007/s00167-008-0507-0 18347778

[25] Maier D Jaeger M Izadpanah K Bornebusch L Suedkamp NP Ogon P. Rotator cuff preservation in arthroscopic treatment of calcific tendinitis. Arthroscopy. (2013) 29(5):824–31. 10.1016/j.arthro.2013.01.031 23566569

[26] Porcellini G Paladini P Campi F Paganelli M. Arthroscopic treatment of calcifying tendinitis of the shoulder: clinical and ultrasonographic follow-up findings at two to five years. J Shoulder Elb Surg. (2004) 13(5):503–8. 10.1016/j.jse.2004.04.001 15383805

[27] Ranalletta M Rossi LA Bongiovanni SL Tanoira I Piuzzi N Maignon G. Arthroscopic removal and rotator cuff repair without acromioplasty for the treatment of symptomatic calcifying tendinitis of the supraspinatus tendon. Orthop J Sports Med. (2015) 3(4):2325967115577957. 10.1177/2325967115577957 26665052 PMC4622339

[28] Marder RA Heiden EA Kim S. Calcific tendonitis of the shoulder: is subacromial decompression in combination with removal of the calcific deposit beneficial? J Shoulder Elb Surg. (2011) 20(6):955–60. 10.1016/j.jse.2010.10.038 21277805

[29] Ark JW Flock TJ Flatow EL Bigliani LU. Arthroscopic treatment of calcific tendinitis of the shoulder. Arthroscopy. (1992) 8(2):183–8. 10.1016/0749-8063(92)90034-9 1637430

[30] Seil R Litzenburger H Kohn D Rupp S. Arthroscopic treatment of chronically painful calcifying tendinitis of the supraspinatus tendon. Arthroscopy. (2006) 22(5):521–7. 10.1016/j.arthro.2006.01.012 16651162

[31] Jerosch J Strauss J Schmiel SJA. Arthroskopische therapie der tendinitis calcarea. Wie wichtig ist die Kalkentfernung. Arthroskopie. (1996) 9:241–5. 10.1007/s001130050078 9082563

[32] Yoo JC Park WH Koh KH Kim SM. Arthroscopic treatment of chronic calcific tendinitis with complete removal and rotator cuff tendon repair. Knee Surg Sports Traumatol Arthrosc. (2010) 18(12):1694–9. 10.1007/s00167-010-1067-7 20151109

[33] Hashiguchi H Iwashita S Okubo A Takai S. Arthroscopic removal and tendon repair for refractory rotator cuff calcific tendinitis of the shoulder. J Nippon Med Sch. (2017) 84(1):19–24. 10.1272/jnms.84.19 28331139

